# California School Staff Reports of Seeing Students Vaping at School and Disciplinary Actions

**DOI:** 10.1177/10598405221127694

**Published:** 2022-09-27

**Authors:** Adam G. Cole, Brianna A. Lienemann, Joanna Sun, Jacqueline Chang, Shu-Hong Zhu

**Affiliations:** 1 Faculty of Health Sciences, 85458 Ontario Tech University, Oshawa, ON, Canada; 2 Moores Cancer Center, 8784 University of California San Diego, La Jolla, CA, USA; 3 The Herbert Wertheim School of Public Health and Human Longevity Science, 8784 University of California San Diego, La Jolla, CA, USA

**Keywords:** vaping, school discipline, adolescent health, alcohol/tobacco/drug use prevention

## Abstract

Youth vaping is a concern and schools may use many approaches to discipline students caught vaping at school. This study identified the prevalence of school staff seeing vaping in schools and the measures used to discipline students. A state-wide sample of 7,938 staff from 255 middle and high schools reported whether they saw any students vaping at school in the last 30 days, whether they have caught any students vaping during class in the last semester, and what happened after catching a student vaping in class. Open-text responses were coded and themes were identified related to disciplinary approaches. 31.9% of staff reported seeing students vaping at school, and 11.9% of teachers reported catching a student vaping during class. Teachers described four categories of disciplinary approaches after catching students vaping in class: no consequences, punitive approaches, restorative approaches, and mixed approaches. Additional support is necessary to help schools address student vaping.

## Introduction

It is widely acknowledged that youth vaping is a prevalent concern in the US (CDC, 2019; Johnston et al., 2021; Levy et al., 2018). The popularity of vaping and the rapidly changing devices present challenges to school staff as students find creative ways to covertly vape at school. Recent evidence indicates that almost two-thirds of middle and high school students in the US reported seeing someone vape in or around their school with the school bathrooms and classrooms being common locations (Dai, 2021; Mantey et al., 2021). Additionally, there are many YouTube videos related to vaping at school, in class, and in the school bathroom (Ramamurthi et al., 2019), demonstrating the pervasiveness of this behavior and the boldness of students. Such frequent exposure to vaping at school can make vaping seem more acceptable, particularly when it is difficult to detect (Allem et al., 2018; Kong et al., 2019; Ramamurthi et al., 2019; Schillo et al., 2020).

Schools can support healthy behaviors, and they are a common, equitable setting for intervention among youth populations since students from a variety of backgrounds spend a large portion of their day at school. School staff, such as principals, teachers, counselors, campus security, and school nurses, are important stakeholders given their daily interactions with students and their involvement in the delivery of substance use prevention education and support. Evidence indicates that the majority of school staff are concerned about students vaping at school, and many feel that preventing vaping is a priority (Schillo et al., 2020). However, despite claims of students increasingly vaping in classrooms and school bathrooms, to our knowledge only a single study has examined the frequency with which school staff have observed this behavior. The study of a small sample of teachers in New Jersey identified that 49% reported seeing someone vaping on school property at least one time in the past 12 months, with 9% seeing someone vaping in class (Fakeh Campbell et al., 2020). There are also a lack of data for what teachers (including instructional aides) report as the consequences when students are caught vaping at school. A variety of disciplinary approaches are possible, including punitive approaches (including calling parents, detention, suspension, or expulsion) and restorative approaches (including counseling or an alternative to suspension program). Such information is necessary to understand the disciplinary approaches that are employed by schools and the supports that may be needed to discourage student vaping at school.

The purpose of this study is to identify school staff experiences with student vaping. Research questions for this study include:

*Research Question 1*: How many school staff report seeing students vaping in school and what are the characteristics of these staff members?
*Research Question 2*: How many teachers report catching a student vaping in class and what are the characteristics of these staff members?
*Research Question 3*: What disciplinary measures are reported by teachers when students are caught vaping in class?

## Methods

### Design and Institutional Review Board Approval

This study used state-wide data collected as part of the 2019–20 cycle of the California Educator Tobacco Survey (CETS, Educator Survey). The CETS is an online, anonymous survey of school staff across California to assess the effectiveness of current tobacco prevention and cessation programs and to provide evidence-based recommendations to California's Department of Education. The survey was administered in conjunction with the California Student Tobacco Survey (CSTS, Student Survey) and gave school staff the opportunity to share their observations and perspectives on student use of tobacco products, vapes, and marijuana to assist in evaluating California's Tobacco-Use Prevention Education (TUPE) Program. The University of California San Diego Human Research Protection Program (#150978) and the California State Committee for the Protection of Human Subjects (#15-07-2105) approved all procedures.

### Setting and Sample Selection

A random selection of schools in California were invited to participate in both the Student and Educator Surveys. Schools were eligible to participate if they were public and nonsectarian schools; schools were excluded if they were special education only, juvenile court schools, district/country community schools, continuation high schools, or other alternative schools. Schools that were only online or that did not have students come to campus at least three days/week were excluded, as were schools that were described as “individualized” or not following a standard curriculum.

As described elsewhere (Zhu et al., 2021), middle schools for the Student and Educator Surveys were sampled using simple state-wide random sampling without stratification by region. Given known geographic differences in tobacco use across California, for the high school sample for the Student and Educator Surveys, the state was divided into 35 regions based on geographic contiguity and cultural similarity, and the number of schools randomly selected within each region proportional to the number of eligible students in the region.

Following completion of the Student Survey, each school was asked to forward an invitation to complete the Educator Survey to school staff who regularly interacted with students (e.g., teachers, counselors, administrators, school nurses). Typically, the invitation was forwarded to staff by the school principal or by a designated contact person. School staff were encouraged to complete the survey within a two-week period, with reminders sent for up to five weeks. School staff could choose whether to enter a draw for a gift card in remuneration for their participation.

A total of 608 schools were invited to participate in the Student Survey between September 2019 and March 2020, prior to the COVID-19 school closures in California (Johnson, 2020). Of the 391 schools that completed the Student Survey, 321 (82.1%) were sent links to complete the Educator Survey between October 2019 and May 2020. From these invited schools, 255 had at least three school staff per school that participated (response rate: 79.4%). An estimated 21,610 school staff were invited to participate and there were 8,378 entries in the dataset. Data were cleaned through a series of steps to remove blank entries (n = 417), remove invalid entries that could not be verified (n = 10), and remove entries from schools with fewer than three participants (n = 13 entries from 10 schools) in order to maintain the anonymity of responses. Therefore 7,938 school staff from 255 schools across California (39 middle schools, 216 high schools) completed the survey (estimated response rate: 36.7%), with an average of 31 school staff per school.

### Measures

The Educator Survey was designed by the researchers to assess school staff knowledge and perceptions of tobacco products (e.g., cigarets, vapes, hookah, little cigars or cigarillos), marijuana, and alcohol and related school prevention and cessation activities. The survey contained 101 unique questions, including topics such as: tobacco prevention and cessation programs at the school, culture of tobacco use in schools, knowledge and attitudes about emerging tobacco products and marijuana, and opinions about the role schools can and should play in tobacco use cessation and prevention. The survey primarily included closed-ended questions, but follow-up open-ended questions allowed participants to provide further details about their selected responses. The survey took an overall median of 17.4 min to complete, was available in English, and used programmed skip logic to reduce participant burden.

Two questions assessed whether school staff had seen students vaping at school. The first was asked of all participants: “In the last 30 days, have you seen any students use vapes at school?” (Yes/No). The second question was only asked of teachers and instructional aides (hereby referred to as “teachers”): “In the last semester, have you caught any students vaping during class?” (Yes/No). Given the lack of evidence for disciplinary consequences for students caught vaping in class, those who reported catching a student vaping during class were asked to describe what happened next. Respondents were also asked to identify their school name, their position at the school, how long they had worked in education, whether part of their role involved providing students with tobacco or other drug use prevention education, whether they had received any training for vape use prevention, intervention, or cessation in the last 3 years, and how much of a problem they thought vaping was for students at their school. These variables were hypothesized to influence whether or not school staff would see or catch students vaping at school.

### Analysis

Descriptive analyses identified the number of school staff who saw students vaping at school in the last 30 days and the number of teachers (including instructional aides) that caught students vaping during class in the last semester, compared across participant characteristics. Two multilevel regression models identified individual- and school-level characteristics associated with (1) school staff reports of seeing students vaping at school in the last 30 days, and (2) teacher reports of catching a student vaping in class in the last semester. Individual-level predictors of interest included the position of the staff member at the school, how long they have worked in education, whether part of their role involved providing students with tobacco or other drug use prevention education, whether they have received any training for vape use prevention, intervention, or cessation in the last 3 years, and how much of a problem they thought vaping was for students at their school. School-level predictors of interest included school type (middle or high school) and region in California. Both models accounted for the clustering of staff within schools and included all predictors. Quantitative analysis were performed using SAS software, Version 9.4 (SAS Institute Inc, 2012).

A step-wise procedure was used to code and analyse the open-text data. First, a random sample of 100 responses were coded by two reviewers (BAL, JC) to create an initial codebook. Next, a random sample of 100 responses were coded by the same two reviewers to calculate the inter-rater reliability (IRR) of coding (pooled Kappa = 0.89). Results of the IRR test were discussed among members of the research team (AGC, BAL, JC) to clarify discrepancies before applying codes to the remaining excerpts. A single individual (BAL) coded the remaining excerpts and identified themes. Dedoose qualitative analysis software (*Dedoose Version 9.0.17*, 2021) was used in the analysis to assist in calculating the IRR and in identifying the major codes and themes.

## Results

The vast majority of participants reported their position as teachers (80.5%), followed by school support, office staff, or other (6.4%), counselors (4.5%), school administrators (3.5%), and instructional aides (3.2%). Only 1.1% of participants reported their position as social workers, school psychologists, or school nurses. Almost half of participants (47.5%) reported working in education for more than 15 years. While 15.9% of participants reported that part of their role at the school was to provide students with tobacco or other drug prevention education, 31.6% reported that it was not part of their role, but they did it anyway.

### Research Question 1: How Many School Staff Report Seeing Students Vaping in School and What Are the Characteristics of These Staff Members?

Almost one third (31.9%) of school staff reported seeing students using vapes at school in the last 30 days. At a school level, almost all schools (94.9% overall, 89.7% of middle schools, 95.8% of high schools) had at least one participant who reported seeing any students vaping at school in the last 30 days. As shown in Table 1, school administrators; campus security, campus supervisors, or TUPE advisors, coordinators, or specialists; and social workers, school psychologists, or school nurses had higher odds of seeing students vaping at school in the last 30 days relative to teachers. The odds of seeing students vaping at school in the last 30 days was also approximately two times higher among those whose role it was to provide tobacco or drug prevention education and who did it even though it was not a part of their role. Those who thought that student vaping was a serious problem at their school had approximately four times greater odds of seeing students vaping at school. School staff from high schools reported higher odds of seeing students vaping at school relative to those from middle schools.

**Table 1.** School Staff Reports of Seeing a Student Vaping at School in the Last 30 Days According to Individual- and School-Level Characteristics, 2019–20 CETS.

Characteristic		Reported seeing a student vaping at school		Odds of seeing a student vaping at school (95% CI)
Participant characteristic		Yes n (%)	No n (%)	
Overall		2531 (31.9)	5407 (68.1)	
Position at the school	Teacher	1651 (27.6)	4327 (72.4)	1.00
	Instructional Aide	70 (29.8)	165 (70.2)	1.33 (0.97, 1.82)
	Counselor	145 (43.8)	186 (56.2)	1.87 (1.47, 2.39)***
	Social Worker, School Psychologist, or School Nurse	45 (50.0)	45 (50.0)	2.20 (1.40, 3.45)***
	School Administrator	221 (84.4)	41 (15.7)	14.21 (9.85, 20.51)***
	Campus Security, Campus Supervisor, or Tobacco Use Prevention Education (TUPE) Advisor, Coordinator, or Specialist	40 (71.4)	16 (28.6)	6.77 (3.57, 12.83)***
	School Support, Office Staff, or Other	196 (41.2)	280 (58.8)	2.13 (1.72, 2.63)***
Length of time worked in education	Less than 1 year	51 (30.7)	115 (69.3)	1.00
	1–5 years	365 (30.7)	826 (69.4)	0.79 (0.54, 1.16)
	6–10 years	376 (30.6)	851 (69.4)	0.81 (0.55, 1.20)
	11–15 years	436 (33.1)	883 (66.9)	0.85 (0.58, 1.24)
	More than 15 years	1141 (32.4)	2384 (67.6)	0.86 (0.59, 1.25)
Part of role to provide students with tobacco or other drug prevention education	No, it's not part of my job	804 (23.2)	2668 (76.8)	1.00
	No, it's not part of my job but I do it anyway	947 (40.4)	1399 (59.6)	2.10 (1.86, 2.38)***
	Yes	521 (44.1)	661 (55.9)	1.98 (1.68, 2.33)***
	I don't know	97 (22.7)	331 (77.3)	1.01 (0.78, 1.30)
Received e-cigarette prevention, intervention, or cessation training in the past 3 years	No	1458 (30.5)	3330 (69.6)	1.00
	Yes	905 (34.4)	1723 (65.6)	0.99 (0.88, 1.12)
How much of a problem do you think vapes are for students in your school?	Not a problem	178 (11.3)	1392 (88.7)	1.00
	Serious problem	2191 (37.5)	3658 (62.5)	4.16 (3.47, 4.97)***
School characteristic				
School type	Middle school	205 (22.6)	701 (77.4)	1.00
	High school	2164 (33.2)	4360 (66.8)	1.40 (1.07, 1.82)*
Region^a^	Northern California	421 (34.4)	802 (65.6)	1.00
	Central California	326 (29.1)	794 (70.9)	0.76 (0.56, 1.04)
	Bay Area	614 (32.5)	1277 (25.2)	0.98 (0.75, 1.29)
	Southern California	1008 (31.5)	2188 (68.5)	0.95 (0.74, 1.21)

*p < 0.05 **p < 0.01 ***p < 0.001.

^a^ The 35 sampling regions were combined into four larger regions to simplify the analyses.

### Research Question 2: How Many Teachers Report Catching a Student Vaping in Class and What Are the Characteristics of These Staff Members?

Among the sample of teachers and instructional aides, approximately one in ten (11.9%) reported catching a student vaping during class in the last semester. At a school level, 78.7% of schools (66.7% of middle schools, 80.6% of high schools) had at least one teacher that reported catching a student vaping during class in the last semester. As shown in Table 2, the odds of catching a student vaping in class in the last semester was higher among teachers whose role it was to provide tobacco or drug prevention education and who did it even though it was not a part of their role. While the odds of catching a student vaping in class was lower among those who received e-cigarette prevention, intervention, or cessation training in the last 3 years, odds were higher among those who thought that student vaping was a serious problem at their school. Teachers from high schools also reported higher odds of catching a student vaping in class relative to those from middle schools.

**Table 2.** Teacher and Instructional Aide Reports of Catching a Student Vaping in Class in the Last Semester According to Individual- and School-Level Characteristics, 2019–20 CETS.

Characteristic		Reported catching a student vaping in class		Odds of catching a student vaping in class (95% CI)
Participant characteristic		Yes n (%)	No n (%)	
Overall		778 (11.9)	5753 (88.1)	
Position at the school	Teacher	753 (12.6)	5248 (87.5)	1.00
	Instructional Aide	25 (10.6)	212 (89.5)	0.92 (0.59, 1.45)
Length of time worked in education	Less than 1 year	13 (8.8)	135 (91.2)	1.00
	1–5 years	129 (13.1)	859 (86.9)	1.38 (0.75, 2.56)
	6–10 years	138 (13.5)	887 (86.5)	1.52 (0.82, 2.81)
	11–15 years	113 (10.6)	956 (89.4)	1.14 (0.61, 2.12)
	More than 15 years	385 (12.8)	2622 (87.2)	1.46 (0.80, 2.67)
Part of role to provide students with tobacco or other drug prevention education	No, it's not part of my job	272 (9.1)	2712 (90.9)	1.00
	No, it's not part of my job but I do it anyway	335 (16.5)	1701 (83.6)	1.84 (1.55, 2.20)***
	Yes	141 (16.7)	703 (83.3)	2.09 (1.66, 2.64)***
	I don't know	30 (8.1)	341 (91.9)	0.94 (0.63, 1.40)
Received e-cigarette prevention, intervention, or cessation training in the past 3 years	No	524 (12.9)	3543 (87.1)	1.00
	Yes	252 (11.7)	1907 (88.3)	0.78 (0.65, 0.93)**
How much of a problem do you think vapes are for students in your school?	Not a problem	54 (3.9)	1323 (96.1)	1.00
	Serious problem	724 (14.9)	4126 (85.1)	3.73 (2.79, 4.98)***
School characteristic				
School type	Middle school	58 (7.4)	724 (92.6)	1.00
	High school	720 (13.2)	4736 (86.8)	1.43 (1.02, 2.01)*
Region^a^	Northern California	165 (15.9)	874 (84.1)	1.00
	Central California	115 (12.2)	829 (87.8)	0.76 (0.54, 1.08)
	Bay Area	182 (11.7)	1370 (88.3)	0.82 (0.61, 1.12)
	Southern California	316 (11.7)	2387 (88.3)	0.76 (0.58, 1.00)

*p < 0.05 **p < 0.01 ***p < 0.001.

^a^ The 35 sampling regions were combined into four larger regions to simplify the analyses.

### Research Question 3: What Disciplinary Measures Are Reported by Teachers When Students Are Caught Vaping in Class?

Teachers and instructional aides who caught students vaping during class were asked to briefly describe what happened next; 93.3% of teachers provided a codeable response. Many teachers described the situation but did not describe any consequences (n = 278). These descriptions helped to illustrate the ubiquity of vaping on some campuses.

“Many students are caught walking around campus vaping or even in the classrooms vaping. Almost a daily occurrence.” (Instructional Aide, high school, Greater Bay Region)

“Walked into the bathroom during my prep and caught some kids selling/buying/using vaping paraphernalia multiple times.” (Teacher, high school, Northern Region)

Many events tended to occur when the teacher was busy teaching the class or speaking with someone else. Teachers noted that students tried to hide their behavior from others, often using their clothing or backpacks to hide the vapor. In these cases, vaping events were reported by other students in the class who noticed the behavior (n = 46).

“Another student told me that he was vaping in my class. The student would just blow [the vapor] down his shirt without me noticing it.” (Teacher, high school, Greater Bay Region)

“A student smoked a vape using a backpack to cover it while I was speaking with a student in the hallway. Another student reported the incident.” (Teacher, high school, Southern Region)

“I did not actually see the student but it was reported by several other students that it happened during a video while the room was dark and the student was on the opposite side of the room.” (Teacher, high school, Northern Region)

Although most students tried to be covert about their vaping, a few teachers also commented on the boldness of some students who vaped in class. These daring occurrences could be captured by other students and posted on social media.

“The student wanted to charge his vape pen during snack. He came in and asked another student out loud if he could borrow their vape pen. I was appalled he didn't have the wherewithal to not be so noticeable.” (Teacher, high school, Southern Region)

“Student had vape pen in their backpack and would lean over and use it periodically in class. Students posted it on Snapchat as students did it in class.” (Teacher, high school, Southern Region)

Some teachers wrote about their frustration with not having adequate education about vaping devices or the noticeable signs that students are vaping in class in order to address the issue. Teachers commented on the fact that because some devices are small and look like a USB drive and some produce very little smell or smoke, it is difficult to know for certain whether a student is vaping in class.

“Apparently the students were challenging themselves to vape in every classroom. I understand that they were successful with this. I didn't realize what was going on at the time because we had had no education ourselves on vaping.” (Teacher, high school, Northern Region)

“I did not know how to respond because I am not familiar with what vaping looks like and did not know what was happening at the time. However, after talking with other teachers, the student was vaping and I regret not responding.” (Teacher, high school, Northern Region)

“Another student informed me this was happening but I had no idea. There was no smoke or smell.” (Teacher, high school, Southern Region)

After describing the situation, one of the first actions taken by many teachers was telling someone else about the incident. This frequently involved reporting the incident and sending the student to administration to be dealt with (n = 291). Teachers frequently noted that this resulted in some kind of disciplinary action, although the description of these consequences was often vague suggesting that teachers are not informed about what happens after the student speaks with an administrator.

“I emailed administration and they called the student up to the office.” (Teacher, high school, Northern Region)

“I discovered them vaping in the restroom around the corner during class time. It was reported and the students were disciplined by the administration.” (Teacher, high school, Southern Region)

“I called our admin, they were searched, and they received some sort of consequence.” (Teacher, high school, Greater Bay Region)

“I told school administration and they took care of it from there.” (Teacher, high school, Northern Region)

In some cases, campus security was called to assist with the issue and to search the student's belongings for vaping devices or paraphernalia (n = 93). There were limitations with calling security as teachers noted that students needed to be caught with devices in order to be penalized. Since students seemed to be aware of this, many of them would discard the device in order to avoid detection and any subsequent consequences.

“A student sat in the back. A puff of smoke was visible. I called security, and they searched him to find a vape device and some THC oil.” (Teacher, high school, Southern Region)

“He passed the pen to someone else before security got here and nothing happened to him because he did not have the pen.” (Teacher, high school, Southern Region)

“After I saw the student vape, I reported the incident to campus security, and the student was taken to the office and searched. Because the student did not have the vaping item on him or in his backpack, he was not punished. The students on my campus know how to get away with vaping at school.” (Teacher, high school, Southern Region)

“I quietly asked the student to meet me off to the side of the volleyball court. I told her I saw her vaping, which she denied. She said, ‘I don't do that! Isn't that like cigarettes?’ Security was called. She later admitted to using the vape pen, which was readily hidden by another student, then found on the volleyball court after class.” (Teacher, high school, Southern Region)

Some teachers described more specific disciplinary approaches when students were caught vaping. These could be grouped into four categories: no consequences, punitive approaches, restorative approaches, and mixed approaches.

#### No consequences

A surprising number of teachers reported no visible consequences for students caught vaping (n = 96). As described previously, this could be because the students did not have a device on them, and since there was no proof they were vaping there could be no consequences. In addition, some teachers noted that it was difficult to identify particular students who were vaping because the vapor was seen in a general area and not around a particular student.

“Saw vapor but couldn't prove anything so nothing happened” (Teacher, high school, Northern Region)

“Just saw a puff of smoke coming from the general area, but couldn't pin point the direct student. Called the office to have them search students. Didn't end up having any consequences because couldn't pin point the student.” (Teacher, high school, Northern Region)

“One of my students last semester vaped in class because there was a big cloud of vape smoke. However, I did not have sufficient evidence to know who was the person who vaped in class.” (Teacher, high school, Southern Region)

Other teachers noted that because of the distinctive smell, they knew someone was vaping but they could not pinpoint which student. Because of this, the students evaded any consequences.

“I smelled the vape but could not identify who did it.” (Teacher, high school, Northern Region)

“I smelled but could not determine who had a vape pen that smelled like marijuana” (Teacher, high school, Southern Region)

“I smelled the sweet vape peach fruity smell at my door during passing period. I continued to smell it, but all students who were near the door equally smelled like that so I couldn't really identify the person or if they were even in my class. I asked about the smell and many students laughed as if they agreed and knew it to be true.” (Teacher, high school, Northern Region)

Some teachers (n = 65) mentioned contacting administration or campus security but nothing happened as a result. This seemed to suggest that there were no clear or consistent consequences for students caught vaping.

“He was reported to the office, but they did nothing about it” (Teacher, high school, Central Region)

“Student used his vape in class during presentations (when the lights were dimmed) I saw the smoke, smelled the vapor, and saw him duck his head to use it. I contacted administration and nothing happened.” (Teacher, high school, Central Region)

“They vaped marijuana, I saw it, they exhaled a big cloud of smoke, were sent to the office, and returned 15 min later, with NO consequences.” (Teacher, high school, Greater Bay Region)

#### Punitive approaches

Punitive approaches are intended to penalize students for their actions as a way of discouraging future infractions, and many teachers (n = 134) reported punitive approaches after catching students vaping in class. They could include low-consequence approaches such as taking the device from the student (n = 48) or contacting the parents (n = 18), and higher-consequence approaches such as detention (n = 2), suspension (n = 49), and in a few cases (n = 4) expulsion. Some teachers (n = 30) reported “disciplinary action” but did not describe what this involved. In a few cases (n = 4), it was reported that the police were involved. Some teachers (n = 20) described multiple punitive approaches, such as contacting parents and suspending students, or taking the device and other disciplinary action. Punitive approaches frequently involved administrators, which was not surprising given their role at the school and ability to penalize students. The following excerpts help to illustrate the range of approaches reported by teachers.

“Right outside of my classroom, I caught a young 9th grader exhaling a big plume of smoke, so I confiscated her device.” (Teacher, high school, Greater Bay Region)

“The student had left the room during class time to ‘go to the bathroom.’ Another student saw vape smoke from a stall and informed an administrator, who talked with the student and his parents.” (Teacher, high school, Greater Bay Region)

“I observed a student with a vape pen on his seat. He then got one or two days in-house punishment (sitting in the school library).” (Teacher, middle school, Southern Region)

“Student had his back to me during a class discussion. I asked him to turn around and face forward. At that point the student coughed and exhaled vape smoke. I called security to have him escorted to the dean's office. The student was suspended for one day.” (Teacher, high school, Central Region)

“The girl put it in her bra so I could not confiscate it. I called the proctor and she was taken to the office and the police were called because the police can search her and school staff cannot.” (Teacher, middle school, Southern Region)

“I sent them to Admin. They were sent to continuation school.” (Teacher, high school, Central Region)

#### Restorative approaches

Restorative approaches are intended to educate students on the harmful effects of vaping to dissuade them from engaging in the behavior. Restorative approaches, which were reported by fewer teachers (n = 46), could involve counseling provided by the teacher or another trained individual. A couple of teachers noted that these opportunities could be used to educate students about the potential harms of vaping:

“I noticed a smell several times that was sweet and students whispering. I then realized where the smell was coming from and counselled the students about dangers of vaping.” (Teacher, middle school, Greater Bay Region)

“The vape was taken to the main office and the student had a discussion with a sports coach about the effects. Administration gave the student a warning.” (Teacher, high school, Northern Region)

The provision of counseling seemed to be in recognition of the fact that vaping could be a coping strategy used by some youth in response to difficult life situations, as illustrated by the following excerpt:

“I took the time to share stories, ask questions, impart education, and listen to them. I wanted the students to articulate what is happening in their personal lives and how those issues are connected to their decision making. I didn't go out of my way to get them in trouble. It was more important for me to use the moment to teach.” (Teacher, high school, Southern Region)

Other restorative approaches included mandating attendance at an alternative to suspension program (n = 23), which seemed to focus on educating students about the harms of vaping. In some cases, teachers noted that students could choose between being suspended or attending an alternative to suspension program. Some teachers noted specific programs that students were assigned to, such as the “Pathways to Success” program or the “7Challenges” program, while others simply noted that students were assigned to an alternative to suspension program. A few teachers (n = 4) also mentioned a “restorative justice” approach but did not provide further details about what this involved.

“The student was referred to the office, searched and a vape was found with a charger and concentrated cannabis oil. The student had been blowing the smoke into his sweatshirt. The student was offered counseling and an alternative to suspension program” (Teacher, high school, Greater Bay Region)

“Referral to admin, vape confiscated, student enrolled in County nicotine program” (Teacher, high school, Northern Region)

One teacher noted a particularly unique program where in addition to an alternative to suspension program, students were also assigned to community service:

“Students are referred to their administrator by being escorted to the office. Students and their family are typically given the option between a suspension or attending the substance use classes at an alternative site where they are educated about the dangers of using such products. They also attend the HOPE school where they do community service and help feed students with severe physical disabilities in hopes of teaching our students how truly blessed they are and to instill the desire to take better physical care of themselves and not take drugs or alcohol or substances such as these.” (Teacher, high school, Southern Region)

#### Mixed approaches

Many teachers (n = 20) who described a punitive approach also described restorative approaches. These mixed approaches tended to involve a penalty, like suspending the student, and requiring attending an alternative education or counseling program.

“They get sent to the office, parents are contacted, they get in school suspension, and have to enroll in a Saturday school vape education course.” (Teacher, high school, Northern Region)

“Security was called and they were escorted from class and suspended in-house. They had to take a class about drug use during that time.” (Teacher, high school, Southern Region)

“I took the vape from the student and took him to the office. He and 5 other students who he said had tried it throughout the day were suspended from school for 2 days and will have 2 days with a counselor talking about nicotine dangers when they return to school.” (Teacher, middle school, Northern Region)

## Discussion

These data from school staff across California highlight the pervasiveness of student vaping at school. Our data indicate that about one third of school staff reported seeing students vaping at school in the past 30 days, and almost all schools had one staff member that reported seeing students vaping at school. Furthermore, about one out of every ten teachers and instructional aides reported catching students vaping in class in the last semester, and the majority of schools had one teacher or instructional aide that reported catching a student vaping in class. Teachers and instructional aides that reported catching a student vaping in class described a range of disciplinary approaches, with punitive approaches being more common than restorative or mixed approaches.

As expected, regression models indicate that compared to teachers, administrators, and student support personnel (such as campus security, counselors, social workers, and school nurses) reported higher odds of seeing students vaping at school. Part of the role of administrators is student discipline, so it is not surprising that they reported higher rates of encountering students vaping at school. Other student support personnel, such as campus security, may be more attuned to students vaping at school given their role at the school. It is noteworthy that counselors, social workers and school nurses also reported higher rates of seeing students vaping and school, as these interactions may provide an opportunity for these types of student support personnel to encourage and support vaping cessation attempts.

There were some differences in individual-level factors associated with both seeing and catching students vaping at school. Compared with those who did not provide tobacco/drug prevention education, school staff whose role it was to provide this prevention education had higher odds of both seeing and catching students vaping at school. Given their knowledge of tobacco products and interactions with students in class, it is likely that these individuals may be more aware of vaping devices and take more notice of vaping on campus. Additionally, those who thought that vaping was a serious problem had higher odds of both seeing and catching students vaping at school. While it is difficult to know for certain based on these cross-sectional data, school staff may report that vaping among students is a more serious problem at their school because they have seen students vaping on campus. Additional longitudinal data are necessary to help clarify the directionality of this association. Finally, our data indicate that school staff from high schools had higher odds of both seeing and catching students vaping at school relative to staff from middle schools. These data reinforce previous studies that identify higher vaping rates among high school students relative to middle school students (Cullen, 2018) and higher rates of exposure to other students vaping at school among high school students relative to middle school students (Dai, 2021; Mantey et al., 2021). However, it remains concerning that the majority of middle schools had at least one staff member that reported seeing or catching a student vaping at school.

The open-text responses provide greater context into student vaping in school and highlight the covert behaviors of students. In the current study, some teachers reported that the small devices and ability to conceal the vapor made it difficult to know whether or not a student was vaping in class. A previous study found that many school staff identified the discreet devices and difficulty in identifying who is vaping as barriers to enforcing vaping policies at school (Schillo et al., 2020). Vaping devices change quickly, and it can be difficult for school staff to remain informed about what popular brands of devices look like; continuous training may be necessary to assist school staff.

In addition, the intersection of the legalization of recreational cannabis in many jurisdictions and novel vaping devices pose additional challenges to school staff. At the time of the study, recreational cannabis was legal for those 21 years of age or older within California, and the prevalence of cannabis use among students in California was higher than that of all other tobacco products combined (Zhu et al., 2021). Therefore, it is possible that some students may have been caught vaping cannabis at school. Cannabis use is associated with motor vehicle crashes and negative health and academic impacts (Hall, 2015; Horwood et al., 2010; National Academies of Sciences, Engineering, and Medicine, 2017). Since it can be difficult for school staff to identify the substance that students are vaping, the Educator Survey did not ask them about the substance students were caught vaping at school.

These data illustrate wide variability in the disciplinary measures employed by schools, which is consistent with previous evidence that indicates significant variation in disciplinary actions across schools and even across classrooms within schools as a result of differences in the tolerance level for misbehavior across teachers (Morrison & Skiba, 2001). While many teachers reported the incident and sent students to administrators, they often provided vague descriptions of disciplinary actions that followed, suggesting that teachers are not sure of the penalties that follow being caught. Notably, some teachers reported no visible consequences when students were caught vaping in class. It is possible that some teachers are less inclined to involve administrators if there are not clear disciplinary actions or protocols to follow when students are caught vaping in school. Previous studies indicate that many school tobacco policies for students are missing a clear description of consequences for violating the policies (Cole et al., 2019), and almost 20% of school staff indicate a lack of clarity about how the school's vaping policy should be enforced (Schillo et al., 2020). Clear communication between administrators and teachers about expectations regarding punitive approaches for student misbehavior can help guide teachers and administrators towards more effective punishment (Collier et al., 2019). Given that clear restrictions regarding tobacco use at school, consistent enforcement of school tobacco policies (Galanti et al., 2014), and stronger school tobacco policies (Cole et al., 2019) are associated with a lower likelihood of tobacco use, school administrators should ensure that there is clear communication with students and school staff about the school's vaping policy, how it will be enforced, and the possible disciplinary actions.

In our sample, more teachers reported punitive approaches for discipline (such as calling parents, student detention or suspension) rather than restorative approaches (such as counseling or alternative to suspension programs) or mixed approaches. Previous studies indicate that there are significant differences in the attitudes of school staff (administrators, teachers, campus police officers) regarding the purpose and function of school disciplinary approaches that affect the type of disciplinary actions (e.g., punitive vs restorative) that are used (Collier et al., 2019; Skiba & Edl, 2004). While some schools may try to blend punitive and restorative approaches, there are numerous barriers including a lack of staff, resources, and time to assist students in a meaningful and individualized way (Collier et al., 2019).

Evidence from parenting research indicates that the level of demandingness and responsiveness are both important for discipline (Baumrind, 1968, 2013). Students will excel when they feel that school rules are strict but fairly enforced (high demandingness) and when they believe that their teachers and other staff members treat them with respect (high responsiveness) (Cornell & Huang, 2016). For example, students at schools that balance these two factors have lower rates of cigarette smoking, alcohol and marijuana use, and bullying (Cornell & Huang, 2016; Gregory et al., 2010; Lau et al., 2018). While it is difficult to judge the level of demandingness and responsiveness in the disciplinary approaches described by teachers in our study, the greater focus on punitive approaches rather than restorative or mixed approaches may suggest that schools are emphasizing demandingness rather than responsiveness. In addition, the lack of description of vaping cessation programs may highlight a missed opportunity for schools to positively intervene with students. While additional evaluation of the impact of these school disciplinary approaches on student vaping is necessary, it is clear that additional guidance is necessary to help schools address student vaping on campus.

### Strengths and Limitations

Strengths of this study include the use of a large, state-wide sample of middle and high schools and school staff from across California. The open-text data provide unique insights into the disciplinary approaches employed when students are caught vaping in class. The structured procedure for coding the open-text data added rigor to the analyses. Limitations include the use of self-reported data which are subject to social desirability bias. The reliability and validity of the measure of seeing vaping on campus is unknown. Although school staff were reminded multiple times to complete the survey, the low participation rate may bias the results. Furthermore, the majority of the sample was teachers, and few were school-based health professionals, such as school nurses. Future studies should examine whether the experiences of other school stakeholders is similar to that of teachers and instructional aides. The Educator Survey did not include questions about participant age, gender, or ethnicity in order to protect the anonymity of participants, particularly in small schools. The exclusion of certain schools from the sample (e.g., continuation schools) that may have higher rates of student substance use limit the generalizability of the findings to these settings. Future studies should explore how the experiences of school staff differ in these types of schools. These data were collected prior to the COVID-19 pandemic. Given evidence that suggests that youth vaping decreased during the early periods of the COVID-19 pandemic when students engaged in remote learning (Kreslake et al., 2021; Leatherdale et al., 2022; Thorisdottir et al., 2021), additional data are needed to investigate whether school staff reports of seeing students vaping on campus have changed. Finally, the survey question did not ask school staff to specify what substance they saw students vaping. Future studies could attempt to differentiate whether disciplinary approaches vary according to the substance students are caught vaping (e.g., nicotine vs THC/CBD).

### Implications for School Nurses

The ubiquity of student vaping on campus provides multiple opportunities for school nurses to work with other staff members to address student vaping. It also highlights the importance of school nurses as part of the school staff team. Given their training and position within the school, school nurses are in a unique position to lead youth vaping prevention and cessation programs for students caught vaping in school. In particular, school nurses can balance the punitive approaches that are already common at many schools with more restorative approaches that seek to educate students about the potential dangers of vaping and counsel them on alternative approaches for dealing with stress and anxiety. School nurses can also work with administrators to ensure there are fair and transparent policies and approaches for dealing with students caught vaping in school.

## Conclusions

These data highlight the ubiquity of student vaping at schools across California. Many school staff reported seeing vaping in school and some teachers reported recently catching students vaping in class. While disciplinary measures varied widely, they tended to focus on punitive rather than restorative approaches. It is likely that school staff need additional training and support in addressing student vaping on campus, and school nurses can play an important role in addressing student vaping.
